# Analysis of ancient mass extinction recoveries in marine environments: generating strategies for managing outcomes of the current mass extinction

**DOI:** 10.1093/nsr/nwad240

**Published:** 2023-09-12

**Authors:** David J Bottjer

**Affiliations:** Department of Earth Sciences, University of Southern California, USA

**Figure 1.**
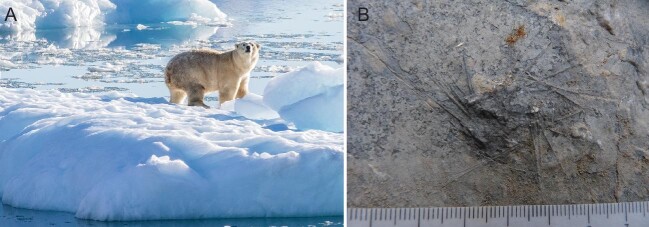
Examples of the role of refugia in surviving mass extinctions. (A) Modern polar bear in southeast Greenland using glacial ice (from [3]—reprinted with permission from AAAS). (B) Earliest Early Triassic fossil sea urchin (echinoid) showing disarticulated spines surrounding the crushed test, which lived in a deeper-water slope setting in what is now China. Scale is in cm/mm. Photograph by Amanda Godbold. Reproduced with permission.

The defining feature of a mass extinction and its associated recovery is the global amount of extinction that occurred [1]. However, there is finer-scale detail to the story, as seen in the ongoing modern mass extinction (the sixth mass extinction), which exhibits variable effects among marine ecosystems and environments, and hence geographic areas [1]. One of the best examples of extreme effects on a broad geographic scale is the plight of modern coral reef ecosystems, which as the ‘canary in the coal mine’ are showing extensive signs of continued devastation [1,2]. Another example of an extreme effect on the broad geographic but individual species scale is the plight of the polar bear, a marine mammal that largely depends upon extensive arctic sea ice to pursue its hunting lifestyle [3]. With increased global warming, arctic sea ice has been disappearing rapidly over recent years, thus imperiling the survival of polar bears. Intriguingly, it has been recently suggested that polar bears may have an enhanced chance of surviving the current mass extinction in nearshore glacial environments of Southeast Greenland, where they hunt from glacial ice (Fig. 1A), which could act as a refugium for the bears [3]. Although much of the possible variability in biotic devastation due to the ongoing modern mass extinction in marine environments and ecosystems remains to be determined, this sort of information will be very relevant in predicting and managing how the oceans will recover once this mass extinction episode subsides.

One way to better understand this biological and spatial variability is to examine ancient mass extinctions, particularly those with the largest amount of extinction (the Big Five mass extinctions [1]), where nature has already run the experiment and we can determine variability due to the mass extinction, and how the recovery proceeded. These mass extinctions are complex intervals in Earth's history, and some of the best cases for geographic and ecosystem variability are from the end-Permian mass extinction and the following Early Triassic, the ∼5-million-year-long recovery period after this mass extinction [4]. The end-Permian mass extinction, the largest of the Big Five ancient mass extinctions affecting marine species (80%–94%), occurred ∼252 million years ago, and was caused by a variety of factors set off by a geologically rapid increase in atmospheric and ocean temperature [5]. This temperature increase was ultimately caused by emplacement and eruption of what is called the Siberian Traps large igneous province [5]. This igneous event involved 2–7 million cubic kilometers of basalt that erupted on the landmass now known as Siberia [5]. Much of the erupting magma passed through carbon-rich sedimentary rocks, the baking of which produced a significant amount of carbon dioxide and hence atmospheric global warming [5]. The geologically sudden global warming caused by this volcanic event is called a hyperthermal, and it can serve as an analogue to our modern global warming, particularly since the modern warming is caused by burning of carbon rich products from sedimentary rock, in this case by our own species [1]. However, a crucial difference is that the modern rate of warming is occurring much faster than that which caused the end-Permian mass extinction (e.g. [1]).

Much like the trend of increasing biotic degradation seen in modern reefs, biotic complexity of Early Triassic reefs was significantly reduced compared with the preceding Late Permian [6]. Lower Triassic reefs are typically relatively small constructions made primarily of microbial features and sponges (e.g. [6]). Reef recovery after the end-Permian mass extinction is characterized by the appearance of scleractinian corals, more than 5 million years later in the Middle Triassic [6]. Similarly, fossils of sea urchins, or echinoids (a group of echinoderms), were strongly affected by the end-Permian mass extinction and are a relatively rare occurrence in the Early Triassic [7]. In marine environments, the global warming associated with this mass extinction shifted the oceanic oxygen-minimum zone onshore, strongly contributing to extinction [4]. Since echinoids are so rare during this recovery interval, it has been of great interest to find an earliest Early Triassic occurrence of echinoids in a deeper-water slope setting (Fig. 1B), which was presumably cooler and with sufficient oxygen, where they survived in what is thought to have been a refugium [8].

As a baseline for the temporal and geographic variability of recovery from this mass extinction, much of the Early Triassic level-bottom shallow seafloor seems to have undergone severe extinction, with only a depauperate fauna, compared to similar seafloor environments before the mass extinction (e.g. [4]). However, several level-bottom environments and locations during this time have been shown to have had a much more diverse and ecologically complex marine fauna (e.g. [9–12]). This confirms that the recovery from the end-Permian mass extinction was temporally, geographically and ecologically patchy, with marine communities in some ecosystems and/or parts of the world recovering more quickly than in others.

These variable biotic, environmental and geographic patterns of recovery from the end-Permian mass extinction can be used as a foundation for a more general model of such variability in mass extinction recovery. Thus, rather than uniform devastation, the Earth is a mosaic of ecosystems and environments, each of which is responding in a different way and at a different rate to the changing environment, caused largely by variability and duration of that mass extinction's trigger and kill mechanisms. Each ecosystem and environment will show different levels of extinction, from mild to extreme. Coming out of a mass extinction the recovery will develop, with these different ecosystems and environments having suffered differing amounts of extinction. Upon this template recovery will ensue, again with this mosaic pattern. Depending upon when the extinction has happened in geological time, varying effects due to differences in paleogeography and ocean chemistry as well as the dominance of different evolutionary faunas, will occur [4].

As this variability has increasingly been documented from the marine Early Triassic, a number of ways of measuring recovery have been utilized. Early Triassic ecological recovery studies have used paleoecological data to establish recovery stages from 1 (least) to 4 (most recovered) (e.g. [11]). Using this approach, different ecosystems at different times in the Early Triassic can be categorized as having recovered on the 1–4 scale (e.g. [11]), and pieces of this mosaic will differentially take a longer or shorter amount of time to recover. Other ways to assess recovery have included using various measures of biodiversity combined with information on degree of ecological complexity [9,10]. From such studies it has been shown that Early Triassic pelagic ecosystems appear to have recovered faster than benthic systems (e.g. [11,12]), and different benthic systems, including those defined by bioturbation, appear to have recovered faster than others [11–13]. In contrast, reef ecosystems show the longest time to recovery (e.g. [6]). It is this mosaic of extinction devastation and subsequent recovery that has led to the variety of recovery schemes utilized for the end-Permian mass extinction, which largely depend upon data type and availability for the ecosystem that is being studied.

Understanding this mosaic and its recovery categorization for the end-Permian mass extinction will be very important for planning and managing the recovery from the sixth mass extinction in modern environments. Establishing which Early Triassic ecosystems in level-bottom environments suffered the most will point the way to modern studies on similar ecosystems and environments. Knowing which modern ecosystems are likely to suffer the most, particularly for marine environments, which have received less attention than terrestrial environments, will allow for establishment of strategies to enable these settings to recover in more rapid fashion [14]. There has already been significant progress in accumulating knowledge on the varying effects of the current mass extinction on different environments and ecosystems (e.g. [14]). Thus, the well-known plight of modern reef ecosystems has stimulated a wide variety of reef conservation biology research (e.g. [2]). Much of this research has focused on the effects of ocean acidification, not only for reefs, but all settings, particularly for open ocean ecosystems (e.g. [1]). Less well-studied are the extensive nearshore and shelf level-bottom environments. In these settings the changing role of bioturbators during such mass extinction stresses is in need of additional attention, as these processes affect not only ecological structure but also biogeochemical processes (e.g. [13]). Comparison of ancient and modern outcomes from mass extinction stresses also needs to filter out additional effects caused by human activity, unique to the modern extinction, such as overfishing, overhunting and global use of pesticides and fertilizers [1,14]. Proceeding in a systematic way, this most important project of conservation paleobiology and paleoecology will allow better management of Earth's ecosystems as mass extinction stresses are diminished. If these modern stresses continue beyond expectations, a very important product of these investigations will be to predict where refuges, such as for the polar bear, might exist for organisms to survive the sixth mass extinction.
